# Supplementary material for: Comparative Genomics of Legionella pneumophila Isolates from the West Bank and Germany Support Molecular Epidemiology of Legionnaires’ Disease
Source: Microorganisms. 2023 Feb 10;11(2):449. doi: 10.3390/microorganisms11020449 (PMC9965269; doi:10.3390/microorganisms11020449)
Supplement: Supplementary file 1 [file microorganisms-11-00449-s001.zip › Supplementary Tables & Figures_Zayed et al.pdf]

Comparative genomics of *Legionella pneumophila* isolates from the West Bank and Germany support molecular epidemiology of Legionnaires' disease

Zayed et al.

SUPPLEMENTARY MATERIALS
TABLES

Table S1: Main features of *L. pneumophila* isolates and reference strains used in the study.

Strain designation	GenBank/ RefSeq Acc. No:	Sg (mAb)[1]	ST[2]	MLVA-8(12)[3]	VACC×	Site of Isolation	Year of Isolation	Source of isolation	Genome Size (bp)	No. of Genes*	Genomic Islands (bp)	Genomic Islands (%)	Coding Seuences*	Contigs	Reason for selection
**Genome sequences obtained within this study**															
A156_Gt64(74)_Ps	0JAPXIV	6 Dresden	ST74	Gt64(74)	VACC2	Beit Jala, West Bank	2013	Hospital environmental swab	3,366,323	3,049	174,331	5.2	3008	40	Thunderbay branch
A129_Gt64(74)_Ps	0JAPXIU	6 Dresden	ST74	Gt64(74)	VACC2	Beit Jala, West Bank	2013	Hospital environmental swab	3,527,232	3,206	293,904	8.3	3166	56	Thunderbay branch
A15_Gt12(84)_Ps	0JAPXIT	Sg.8	ST 1358	Gt12(84)	NA	East Jerusalem, West Bank	2012	Hospital environmental swab	3,495,260	3,088	180,013	5.2	3,053	127	Lorraine branch
A139_Gt4(17)_Ps	0JAPXIS	Sg.1	ST1	Gt4(17)	VACC1	Nablus, West Bank	2013	Hospital environmental swab	3,579,958	3,242	223,851	6.3	3,205	78	Worldwide present and highest abundance in our collections
A5_Gt4(17)_Ps	0JAPXIR	1 OLDA	ST1	Gt4(17)	VACC1	East Jerusalem, West Bank	2012	Hospital environmental swab	3,586,669	3,249	225,997	6.3	3,211	80	Worldwide present and highest abundance in our collections

A29_Gt6(18)_Ps	0JAPXIQ	Sg.1	ST1	Gt6(18)	VAC C1	Hebron, West Bank	2012	Hospital environmental swab	3,626,550	3,289	339,005	9.3	3,251	68	Dominant in southern West Bank
A131_Gt6(18)_Ps	0JAPXIP	Sg.1	ST1	Gt6(18)	VAC C1	Hebron, West Bank	2013	Hospital environmental swab	3,548,746	3,206	281,219	7.9	3,169	67	Dominant in southern West Bank
A193_Gt40(47)_Ps	0JAPXIO	6 Dresden	ST345	Gt40(47)	VAC C5	Hebron, West Bank	2014	Hospital environmental swab	3,446,379	3,098	185,152	5.4	3,056	33	Alcoy branch, close to Warstein outbreak
A194_Gt40(47)_Ps	CP114578	6 Dresden	ST345	Gt40(47)	VAC C5	Hebron, West Bank	2014	Hospital environmental swab	3,467,904	3,121	156,728	4.5	3,071	1	Alcoy branch, close to Warstein outbreak
A195_Gt40(47)_Ps	JAPXIN	6 Dresden	ST345	Gt40(47)	VAC C5	Hebron, West Bank	2014	Hospital environmental swab	3,440,802	3,095	167,241	4.9	3,051	42	Alcoy branch, close to Warstein outbreak
A138_Gt9(92)_Ps	JAPXIM	6 Dresden	ST461	Gt9(92)	VAC C11	Nablus, West Bank	2013	Hospital environmental swab	3,363,143	3,038	162,571	4.8	2,996	40	Alcoy branch
A112_Gt10(93)_Ps	JAPXIL	6 Dresden	ST461	Gt10(93)	VAC C11	Beit Jala, West Bank	2013	Hospital environmental swab	3,362,286	3,030	147,968	4.4	2,990	42	Highly abundant genotype
A114_Gt10(93)_Ps	JAPXIK	6 Dresden	ST461	Gt10(93)	VAC C11	Beit Jala, West Bank	2013	Hospital hot water sample	3,362,313	3,033	159,647	4.7	2,989	41	Highly abundant genotype
A127_Gt10(93)_Ps	JAPXIJ	6 Dresden	ST461	Gt10(93)	VAC C11	Beit Jala, West Bank	2013	Hospital environmental swab	3,363,025	3,037	142,978	4.3	2,993	38	Highly abundant genotype
A108_Gt10(93)_Ps	JAPXII	6 Dresden	ST461	Gt10(93)	VAC C11	Beit Jala, West Bank	2013	Hospital environmental swab	3,291,620	2,949	153,342	4.7	2,907	38	Highly abundant genotype
A166_Gt8(142)_Ps	JAPXIH	Sg.(2-14)	ST1482	Gt8(142)	VAC C5	Hebron, West Bank	2013	Hospital environmental swab	3,353,128	3,014	204,232	6.1	2,974	28	Alcoy branch
H34_Gt22(102)_D	JAPXIG	Sg.10	NA	Gt22(102)	NA	Braunschweig, Germany	2013	Environmental sample	3,403,136	3,049	160,329	4.7	3,016	64	D7630 branch

H29_Gt22(100)_D	JAPXIF	4 Portland	NA	Gt22(100)	VAC C2	Braunschweig, Germany	2013	Environmental sample	3,403,391	3,061	144,415	4.2	3,017	68	D7630 branch
H35_Gt22(102)_D	JAPXIE	Sg.1	NA	Gt22(102)	VAC C5	Braunschweig, Germany	2013	Environmental sample	3,402,267	3,049	166,533	4.9	3,013	64	D7630 branch
H39_Gt4(17)_D	JAPXID	4 Portland	NA	Gt4(17)	NA	Braunschweig, Germany	2014	Environmental sample	3,592,274	3,245	226,268	6.3	3,202	78	Worldwide present and highest abundance in our collections
H3_Gt14(31)_D	CP114576, CP114577	6 Chicago	ST1431	Gt14(31)	VAC C2	Braunschweig, Germany	2009	Environmental sample	3,691,263	3,386	209,867	5.7	3,336	2	Alcoy branch
H23_Gt14(30)_D	JAPXIC	6 Chicago	ST1431	Gt14(30)	VAC C2	Braunschweig, Germany	2013	Environmental sample	3,783,853	3,482	372,750	9.9	3,444	130	Alcoy branch
H1_Gt14(31)_D	JAPXIB	6 Chicago	ST1431	Gt14(31)	VAC C2	Braunschweig, Germany	2009	Environmental sample	3,654,038	3,355	253,073	6.9	3,312	69	Alcoy branch
H2_Gt14(31)_D	JAPXIA	6 Chicago	ST1431	Gt14(31)	VAC C2	Braunschweig, Germany	2009	Environmental sample	3,650,216	3,347	238,649	6.5	3,310	64	Alcoy branch
L09-313_Cl_Gt84(116)_D	JAPXHZ	Sg.3	ST93	Gt84(116)	VAC C13	Freiburg, Germany	2009	Clinical sample	3,398,240	3,068	160,181	4.7	3,027	58	Thunderbay branch
L01-443_Cl_Gt64(74)_D	JAPXHY	1 Knoxville	ST9	Gt64(74)	VAC C13	Herford, Germany	2001	Clinical sample	3,477,581	3,142	225,639	6.5	3,099	103	Thunderbay branch
L10-091_Cl_Gt69(69)_D	JAPXHX	1 Philadelphia	ST435	Gt69(69)	VAC C2	Erlangen, Germany	2010	Clinical sample	3,687,459	3,373	187,440	5.1	3,330	97	Pontiac Branch
L12-317_Cl_Gt72(66)_D	JAPXHW	1 Knoxville	ST444	Gt72(66)	VAC C2	Lorrach, Germany	2012	Clinical sample	3,516,737	3,170	186,733	5.3	3,133	74	Pontiac Branch
L11-209_Cl_Gt29(27)_D	JAPXHV	1 Knoxville	ST62	Gt29(27)	VAC C2	Hannover, Germany	2011	Clinical sample	3,461,996	3,130	96,408	2.8	3,085	65	Pontiac Branch
L10-023_Cl_Gt75(49)_D	CP011105, CP011106	1 Knoxville	ST62	Gt75(49)	VAC C2	Ulm, Germany	2010	Clinical sample	3,641,882	3,427	NA	NA	3,377	1	Pontiac Branch

L09-329_Cl_Gt75(49)_D	JAPXHU	1 Philadelphia	ST62	Gt75(49)	VAC C2	Trier, Germany	2009	Clinical sample	3,445,544	3,108	117,416	3.4	3,068	76	Pontiac Branch
L02-521_Cl_Gt29(27)_D	JAPXHT	1 Philadelphia	ST62	Gt29(27)	VAC C2	Bad Langensalza, Germany	2002	Clinical sample	3,478,585	3,161	118,405	3.4	3,117	72	Pontiac Branch
L02-465_Cl_Gt27(133)_D	JAPXHS	1 Benidorm	ST425	Gt27(133)	VAC C1	Berlin, Germany	2002	Clinical sample	3,500,734	3,173	214,498	6.1	3,135	52	Alcoy branch
L04-545_Cl_Gt40(47)_D	JAPXHR	6 Dresden	ST292	Gt40(47)	VAC C2	Heide-West, Germany	2004	Clinical sample	3,358,959	3,006	160,642	4.8	2,964	34	Alcoy branch, close to Warstein outbreak
L06-153_Cl_Gt71(135)_D	JAPXHQ	1 OLDA	ST169	Gt71(135)	VAC C18	Brandenburg , Germany	2006	Clinical sample	3,405,980	3,074	NA	NA	3,030	39	Alcoy branch
L06-129_Cl_Gt71(135)_D	JAPXHP	1 OLDA	ST169	Gt71(135)	VAC C18	Brandenburg , Germany	2006	Clinical sample	3,406,112	3,075	172,495	5.1	3,031	40	Alcoy branch
L04-041_Cl_Gt30(137)_D	JAPXHO	Sg.3	ST87	Gt30 (137)	NA	Kassel, Germany	2004	Clinical sample	3,522,671	3,206	258,174	7.3	3,165	50	Alcoy branch
L05-341_Cl_Gt8(132)_D	JAPXHN	6 Chicago	ST81	Gt8(132)	VAC C16	Darmstadt, Germany	2005	Clinical sample	3,410,856	3,071	219,558	6.4	3,035	31	Alcoy branch
**Reference genomes used within this study**															
Lpn-LPE509	CP003886	NA	NA	NA	NA	Shanghai, China	2013	Hospital water	3,434,224	3,105	NA	NA	3,055	1	Already published
Lpn-Philadelphia1	NC_002942	1 Philadelphia	ST36	Gt64(74)	VAC C2	Philadelphia, USA	1974	Clinical sample	3,397,754	2,943	180,555	5.3	3,023	1	Type strain-well characterized/ cause Philadelphia outbreak/ Already published
Lpn-Thunderbay	CP003730	Sg.6	ST187	NA	NA	Thunderbay, Canada	Unknown	Clinical sample	3,455,167	2,998	NA	NA	3,116	1	Already published

Lpn-ATCC43290	CP003192	Sg.12	ST187	NA	NA	USA	1987	Clinical sample	3,359,001	2,926	NA	NA	2,993	1	Already published
Lpn-lpm7613	NZ_LT598657, NZ_LT598658	NA	NA	NA	NA	NA	2000	Clinical sample	3,261,562	2,944	NA	NA	2,894	1	Already published
Lpn-Lens	CR628337, CR628339	Sg.1	ST15	NA	NA	France	2004	Clinical sample	3,345,687	3,004	180,986	5.4	2,956	1	Caused a large outbreak in France/ already published
Lpn-Lorraine	FQ958210	Sg.1	ST47	NA	NA	France	2004	Clinical sample	3,467,254	3,069	NA	NA	3,130	1	In top six strains that cause disease/ already published
Lpn-HL06041035	FQ958211	NA	NA	NA	NA	France	2006	Hospital water	3,492,535	3,184	NA	NA	3,113	1	Already published
Lpn-D7631	CP015343	Sg.1	ST731	NA	NA	NYC, USA	2012	Environmental sample	3,436,178	3,090	NA	NA	3,040	1	Already published
Lpn-D7630	CP015344	Sg.1	ST731	NA	NA	NYC, USA	2012	Clinical sample	3,444,702	3,097	NA	NA	3,047	1	Already published
Lpn-D7632	CP015342	Sg.1	ST731	NA	NA	NYC, USA	2012	Clinical sample	3,435,648	3,087	NA	NA	3,037	1	Already published
Lpn-Paris	CIP107629	1 Philadelphia	ST1	Gt4(17)	VAC C1	Paris, France	2004	Clinical sample	3,503,610	3,224	163,637	4.7	3,101	1	Worldwide distributed/ already published/ manually annotated
Lpn-OLDA	CP016030	1 OLDA	ST1	NA	NA	USA	1947	Frozen cell culture	3,486,108	3,144	NA	NA	3,094	1	already published
Lpn-Pontiac	CP016029	1 OLDA	NA	NA	NA	Michigan, USA	1968	Sporadic LD case	3,545,001	3,224	NA	NA	3,174	1	Already published
Lpn-Toronto	CP012019.1	NA	NA	NA	NA	Toronto, Canada	2005	Clinical sample	3,573,898	3,269	NA	NA	3,187	1	Already published

Lpn-Alcoy 2300/99	CP001828	Sg.1	ST578	NA	NA	Alcoy, Spain	1999	Clinical sample	3,516,334	3,190	157,442	4.5	NA	1	Caused big outbreak in Spain/ already published/ manually annotated
Lpn-Corby	CP000675	1 Knoxville	ST1685 or ST51	Gt86(97)	NA	UK	1985	Clinical sample	3,576,470	3,204	217,089	6.1	NA	1	already published/manually annotated
¹ Serogroup (monoclonal antibody)			² Sequence Type		³ Multi-Locus of Variable Number of Tandem Repeats Analysis using 13 loci				× VNTR-Analysis -Clonal Complex				* Based on Prokka annotation		

Table S2: The dDDH values among all *L. pneumophila* isolates provided according to TYGS. (Provided in Excel file)

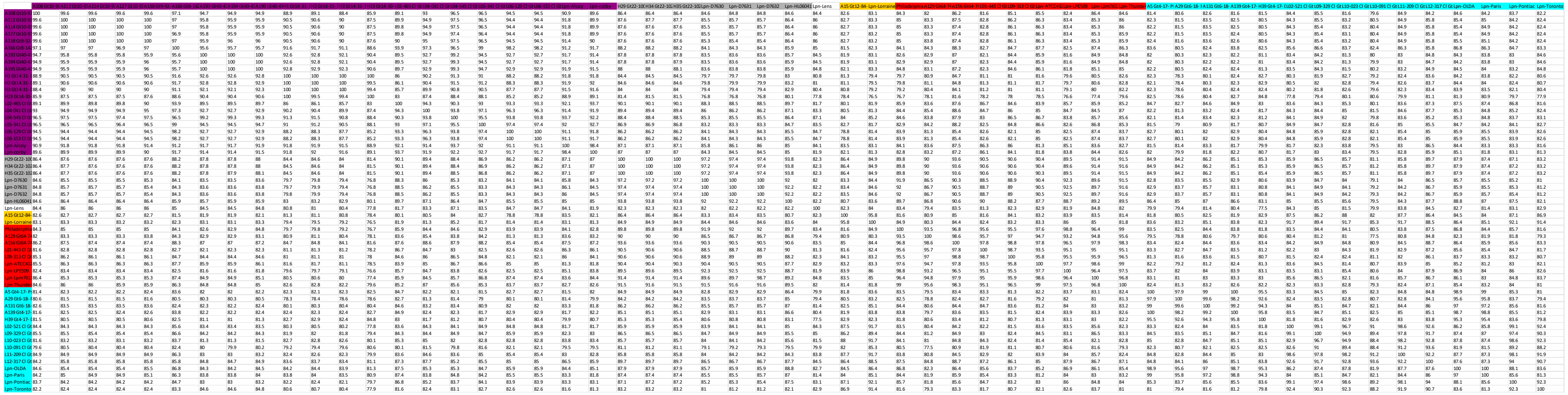

Table S3: Whole-genome SNP comparison of *L. pneumophila* isolates and reference strains. Numbers indicate SNP different from the reference genome

***L. pneumophila* strain**	**Lpn-LPE509**	**Lpn-Phildelphia1**	**Lpn-Thunderbay**	**Lpn-ATCC43290**	**Lpn-lpm7613**	**Lpn-Lens**	**Lpn-Lorraine**	**Lpn-HL06041035**	**Lpn-D7631**	**Lpn-D7630**	**Lpn-D7632**	**Lpn-Paris**	**Lpn-OLDA**	**Lpn-Pontiac**	**Lpn-Toronto**	**Lpn-Alcoy**	**Lpn-Corby**	**A194-Gt40(47)-Ps**	**H3-Gt14(31)-D**
Lpn-LPE509	0	7,893	6,607	4,432	5,681	61,660	52,372	55,729	51,231	51,231	51,232	54,130	54,189	54,867	57,050	56,175	56,022	56,954	56,967
Lpn-Phildelphia1	7,949	0	7,173	6,931	8,114	60,513	52,197	57,012	54,113	54,113	54,114	56,508	56,567	57,130	58,655	59,954	59,707	59,815	59,716
L09-313_Cl_Gt84(116)_D	7,497	6,725	**3,463**	3,736	4,614	60,800	52,184	58,590	52,244	52,244	52,245	59,151	59,210	57,112	58,850	56,022	56,099	56,924	57,071
L01-443_Cl_Gt64(74)_D	9,711	8,315	**6,283**	6,589	7,259	60,203	52,162	58,289	52,019	52,019	52,020	59,314	59,373	57,215	59,036	56,337	56,576	57,346	57,053
Lpn-Thunderbay	6,645	7,122	0	2,376	3,271	60,903	52,499	57,766	50,688	50,688	50,689	58,421	58,480	57,208	58,841	56,525	56,277	57,458	57,151
Lpn-ATCC43290	4,471	6,876	2,376	0	1,663	61,037	52,710	57,620	50,680	50,680	50,681	58,102	58,161	56,886	58,502	56,719	56,690	57,633	57,324
Lpn-lpm7613	5,727	8,062	3,270	1,665	0	60,217	52,715	57,852	51,426	51,426	51,427	58,455	58,518	57,005	58,682	56,692	56,664	57,919	57,438
A156_Gt64(74)_Ps	6,255	5,700	**3,018**	3,288	4,185	61,079	52,328	58,651	50,720	50,720	50,721	59,348	59,407	57,387	58,767	55,986	55,492	57,772	56,644
A129_Gt64(74)_Ps	6,251	5,700	**3,018**	3,288	4,185	61,078	52,328	58,651	50,720	50,720	50,721	59,348	59,407	57,387	58,767	55,986	55,492	57,772	56,644
Lpn-Lens	62,174	60,985	61,462	61,608	60,755	0	55,607	68,139	66,603	66,603	66,604	67,884	67,917	62,288	63,962	70,708	71,182	70,922	70,076

Lpn-Lorraine	52,252	52,094	52,408	52,634	52,624	54,968	0	50,857	52,019	52,019	52,020	46,520	46,547	40,566	44,745	53,380	53,848	53,596	53,236
A15_Gt12(84)_Ps	53,544	52,817	52,966	53,275	53,208	52,506	**13,180**	53,553	54,043	54,043	54,044	50,895	50,922	48,035	49,236	55,931	56,527	56,183	55,605
Lpn-HL06041035	55,334	56,644	57,431	57,284	57,502	66,966	50,330	0	28,898	28,898	28,899	32,907	32,968	39,435	40,855	47,898	48,866	48,578	45,746
Lpn-D7631	50,817	53,705	50,323	50,325	51,070	65,514	51,655	29,003	0	0	1	43,822	43,887	46,214	46,966	45,673	46,299	46,044	44,305
Lpn-D7630	50,817	53,705	50,323	50,325	51,070	65,514	51,655	29,003	0	0	1	43,822	43,887	46,214	46,966	45,673	46,299	46,044	44,305
Lpn-D7632	50,818	53,706	50,324	50,326	51,071	65,515	51,656	29,004	1	1	0	43,823	43,888	46,215	46,967	45,674	46,300	46,045	44,306
H34_Gt22(102)_D	53,856	58,095	54,717	54,848	54,850	66,807	51,795	25,429	**9,793**	**9,793**	9,794	43,034	43,095	44,852	45,631	43,272	43,761	44,291	41,670
H29_Gt22(100)_D	53,855	58,066	54,688	54,819	54,821	66,778	51,766	25,400	**9,764**	**9,764**	9,765	43,005	43,066	44,823	45,602	43,243	43,732	44,262	41,641
H35_Gt22(102)_D	53,855	58,132	54,750	54,885	54,887	66,844	51,832	25,466	**9,831**	**9,831**	9,832	43,071	43,132	44,889	45,669	43,310	43,799	44,329	41,708
H39_Gt4(17)_D	55,366	57,545	57,882	57,756	57,893	62,815	47,911	36,501	44,709	44,709	44,710	11,084	**11,020**	32,760	32,365	46,510	46,595	46,619	46,258
A139_Gt4(17)_Ps	53,876	56,316	58,267	57,951	58,294	66,870	46,172	33,110	43,893	43,893	43,894	260	**195**	28,098	29,498	46,344	46,958	46,574	46,185
Lpn-Paris	53,754	56,173	58,128	57,812	58,155	66,767	46,068	32,935	43,744	43,744	43,745	0	90	28,057	29,457	46,188	46,802	46,417	46,033
A5_Gt4(17)_Ps	53,881	56,289	58,244	57,928	58,271	66,851	46,147	33,054	43,863	43,863	43,864	147	**76**	28,080	29,480	46,305	46,919	46,534	46,150
Lpn-OLDA	53,817	56,232	58,187	57,871	58,214	66,794	46,092	32,997	43,807	43,807	43,808	89	0	28,026	29,424	46,247	46,861	46,476	46,092
A29_Gt6(18)_Ps	53,003	56,351	57,396	57,080	57,411	66,653	46,114	32,784	42,711	42,711	42,712	1,753	**1,688**	28,114	29,416	45,065	45,636	45,379	44,982
A131_Gt6(18)_Ps	53,001	56,347	57,392	57,076	57,407	66,651	46,112	32,782	42,709	42,709	42,710	1,751	**1,686**	28,112	29,414	45,061	45,632	45,375	44,978

L10-091_Cl_Gt69(69)_D	54,313	56,966	57,043	56,697	56,892	64,690	42,654	38,454	44,541	44,541	44,542	24,407	24,377	**14,358**	15,657	45,825	45,796	46,448	46,077
L12-317_Cl_Gt72(66)_D	54,279	56,915	56,951	56,621	56,801	64,191	42,018	38,499	44,954	44,954	44,955	25,422	25,392	**14,949**	16,587	45,264	45,257	46,004	45,589
L11-209_Cl_Gt29(27)_D	54,688	56,911	56,569	56,259	56,280	60,112	40,762	40,236	46,186	46,186	46,187	28,696	28,667	**2,704**	17,498	47,311	47,981	47,912	47,050
L10-023_Cl_Gt75(49)_D	54,483	56,779	56,797	56,483	56,586	61,117	40,207	39,537	46,013	46,013	46,014	27,898	27,869	**823**	16,393	46,774	47,339	47,539	46,453
L09-329_Cl_Gt75(49)_D	54,327	56,492	56,579	56,342	56,445	59,975	40,316	40,251	46,761	46,761	46,762	29,248	29,219	**2,329**	17,797	47,545	47,965	48,201	47,333
Lpn-Pontiac	54,350	56,657	56,744	56,430	56,533	61,016	39,951	39,432	45,999	45,999	46,000	27,956	27,927	0	16,484	46,973	47,501	47,597	46,747
L02-521_Cl_Gt29(27)_D	54,215	56,521	56,608	56,294	56,397	60,051	40,083	40,073	46,572	46,572	46,573	29,082	29,053	**2,030**	17,670	47,787	48,273	48,363	47,595
Lpn-Toronto	56,626	58,242	58,470	58,127	58,294	62,885	44,296	40,967	46,900	46,900	46,901	29,477	29,446	16,653	0	46,576	47,001	47,176	46,853
L02-465_Cl_Gt27(133)_D	56,947	59,635	57,486	57,654	57,718	67,881	51,844	43,966	39,939	39,938	39,939	43,405	43,466	45,846	46,267	19,764	20,122	**19,031**	21,146
L04-545_Cl_Gt40(47)_D	56,226	59,148	56,602	56,796	57,088	69,342	52,781	48,072	46,337	46,336	46,337	45,808	45,869	47,918	46,700	11,776	12,448	**2,822**	15,112
A193_Gt40(47)_Ps	56,790	59,399	57,082	57,269	57,542	69,601	53,182	48,816	46,127	46,126	46,127	46,656	46,717	48,220	47,519	13,008	14,269	**76**	16,883
A194_Gt40(47)_Ps	55,980	58,832	56,516	56,703	56,976	69,038	52,613	48,246	45,547	45,546	45,547	46,086	46,147	47,651	46,952	12,623	13,696	0	16,319

A195_Gt40(47)_Ps	56,044	58,896	56,580	56,767	57,040	69,101	52,677	48,309	45,612	45,611	45,612	46,150	46,211	47,715	47,016	12,686	13,759	**69**	16,383
A138_Gt9(92)_Ps	55,387	58,320	56,257	56,577	56,771	69,093	52,304	47,447	46,595	46,594	46,595	45,200	45,262	47,414	47,230	11,674	12,977	**9,235**	15,497
A112_Gt10(93)_Ps	55,391	58,287	56,228	56,579	56,773	69,035	52,119	47,331	46,562	46,561	46,562	45,023	45,085	47,006	46,832	12,080	13,387	**9,737**	15,668
A114_Gt10(93)_Ps	55,397	58,293	56,234	56,585	56,779	69,041	52,125	47,337	46,568	46,567	46,568	45,029	45,091	47,012	46,838	12,086	13,393	**9,743**	15,674
A127_Gt10(93)_Ps	55,398	58,324	56,265	56,616	56,810	69,073	52,157	47,369	46,600	46,599	46,600	45,061	45,123	47,044	46,870	12,119	13,426	**9,776**	15,707
A108_Gt10(93)_Ps	55,400	58,296	56,237	56,588	56,782	69,044	52,128	47,340	46,571	46,570	46,571	45,032	45,094	47,015	46,841	12,089	13,396	**9,746**	15,677
H3_Gt14(31)_D	55,599	58,312	55,793	55,969	56,073	67,765	51,960	45,184	43,502	43,501	43,502	45,499	45,560	46,694	46,414	12,709	13,932	15,997	0
H23_Gt14(30)_D	55,605	58,318	55,799	55,975	56,079	67,771	51,966	45,190	43,508	43,507	43,508	45,505	45,566	46,700	46,420	12,715	13,938	16,003	**8**
H1_Gt14(31)_D	55,602	58,315	55,796	55,972	56,076	67,768	51,963	45,187	43,505	43,504	43,505	45,502	45,563	46,697	46,417	12,712	13,935	16,000	**5**
H2_Gt14(31)_D	55,602	58,315	55,796	55,972	56,076	67,768	51,963	45,187	43,505	43,504	43,505	45,502	45,563	46,697	46,417	12,712	13,935	16,000	**5**
Lpn-Alcoy	55,191	58,936	55,551	55,754	55,715	68,796	52,373	47,589	45,173	45,172	45,173	45,889	45,950	47,059	46,349	0	8,536	12,575	12,992
Lpn-Corby	54,760	58,420	55,226	55,457	55,428	69,105	52,667	48,358	45,614	45,613	45,614	46,311	46,372	47,418	46,605	8,569	0	13,669	14,240
L06-153_Cl_Gt71(135)_D	56,435	60,030	56,786	57,052	57,085	70,197	53,883	49,343	46,579	46,578	46,579	46,688	46,749	48,436	47,569	11,960	12,203	**11,755**	15,637
L06-129_Cl_Gt71(135)_D	56,432	60,027	56,783	57,049	57,082	70,194	53,880	49,340	46,576	46,575	46,576	46,685	46,746	48,433	47,566	11,957	12,200	**11,752**	15,634
A166_Gt8(142)_Ps	56,734	59,717	57,005	57,273	57,506	69,648	53,331	48,134	44,949	44,948	44,950	45,390	45,451	47,608	46,775	13,650	13,572	**11,522**	16,010

L04-041_Cl_Gt30(137)_D	55,304	58,994	55,498	55,768	55,990	68,718	52,568	45,987	42,328	42,327	42,328	45,102	45,163	46,619	46,345	14,500	14,015	**<u>13,889</u>**	15,584
L05-341_Cl_Gt8(132)_D	56,279	59,240	56,383	56,663	56,758	69,406	52,920	47,086	45,076	45,075	45,076	45,359	45,418	47,058	46,395	13,146	13,809	**<u>11,468</u>**	14,975
**Sum of SNPs**	2,578,310	2,701,132	2,617,760	2,616,399	2,630,850	3,523,725	2,659,278	2,398,444	2,314,928	2,314,907	2,314,961	2,175,712	2,177,492	2,223,081	2,313,226	**<u>1,930,136</u>**	1,983,096	1,943,366	1,962,137
A color represents relative number of SNPs differences. Each branch has specific color code as given in Figure 1. **Bold** and <u>Underlined</u> = Least No. of SNPs																			

Table S4: Genome-wide quantification of SNPs against *L. pneumophila* strain Alcoy					
L. pneumophila isolates	SNPs count (bp)	SNPs count (Mbp)	Genome size (bp)	Genome size (Mbp)	SNPs/Mbp
Lpn-LPE509	56,175	0.056	3,434,224	3.43	0.016
Lpn-Phildelphia1	59,954	0.060	3,397,754	3.40	0.018
L09-313_Cl_Gt84(116)_D	56,022	0.056	3,398,240	3.40	0.016
L01-443_Cl_Gt64(74)_D	56,337	0.056	3,477,581	3.48	0.016
Lpn-Thunderbay	56,525	0.057	3,455,167	3.46	0.016
Lpn-ATCC43290	56,719	0.057	3,359,001	3.36	0.017
Lpn-lpm7613	56,692	0.057	3,261,562	3.26	0.017
A156_Gt64(74)_Ps	55,986	0.056	3,366,323	3.37	0.017
A129_Gt64(74)_Ps	55,986	0.056	3,527,232	3.53	0.016
Lpn-Lens	70,708	0.071	3,345,687	3.35	0.021
Lpn-Lorraine	53,380	0.053	3,467,254	3.47	0.015
A15_Gt12(84)_Ps	55,931	0.056	3,495,260	3.50	0.016
Lpn-HL06041035	47,898	0.048	3,492,535	3.49	0.014
Lpn-D7631	45,673	0.046	3,436,178	3.44	0.013
Lpn-D7630	45,673	0.046	3,444,702	3.44	0.013
Lpn-D7632	45,674	0.046	3,435,648	3.44	0.013
H34_Gt22(102)_D	43,272	0.043	3,403,136	3.40	0.013
H29_Gt22(100)_D	43,243	0.043	3,403,391	3.40	0.013
H35_Gt22(102)_D	43,310	0.043	3,402,267	3.40	0.013
H39_Gt4(17)_D	46,510	0.047	3,592,274	3.59	0.013
A139_Gt4(17)_Ps	46,344	0.046	3,579,958	3.58	0.013
Lpn-Paris	46,188	0.046	3,503,610	3.50	0.013
A5_Gt4(17)_Ps	46,305	0.046	3,586,669	3.59	0.013
Lpn-OLDA	46,247	0.046	3,486,108	3.49	0.013
A29_Gt6(18)_Ps	45,065	0.045	3,626,550	3.63	0.012
A131_Gt6(18)_Ps	45,061	0.045	3,548,746	3.55	0.013
L10-091_Cl_Gt69(69)_D	45,825	0.046	3,687,459	3.69	0.012
L12-317_Cl_Gt72(66)_D	45,264	0.045	3,516,737	3.52	0.013

L11-209_Cl_Gt29(27)_D	47,311	0.047	3,461,996	3.46	0.014
L10-023_Cl_Gt75(49)_D	46,774	0.047	3,641,882	3.64	0.013
L09-329_Cl_Gt75(49)_D	47,545	0.048	3,445,544	3.45	0.014
Lpn-Pontiac	46,973	0.047	3,545,001	3.55	0.013
L02-521_Cl_Gt29(27)_D	47,787	0.048	3,478,585	3.48	0.014
Lpn-Toronto	46,576	0.047	3,573,898	3.57	0.013
L02-465_Cl_Gt27(133)_D	19,764	0.020	3,500,734	3.50	0.006
L04-545_Cl_Gt40(47)_D	11,776	0.012	3,358,959	3.36	0.004
A193_Gt40(47)_Ps	13,008	0.013	3,446,379	3.45	0.004
A194_Gt40(47)_Ps	12623	0.013	3467904	3.47	0.004
A195_Gt40(47)_Ps	12,686	0.013	3,440,802	3.44	0.004
A138_Gt9(92)_Ps	11,674	0.012	3,363,143	3.36	0.003
A112_Gt10(93)_Ps	12,080	0.012	3,362,286	3.36	0.004
A114_Gt10(93)_Ps	12,086	0.012	3,362,313	3.36	0.004
A127_Gt10(93)_Ps	12,119	0.012	3,363,025	3.36	0.004
A108_Gt10(93)_Ps	12,089	0.012	3,291,620	3.29	0.004
H3_Gt14(31)_D	12709	0.013	3,691,263	3.69	0.003
H23_Gt14(30)_D	12,715	0.013	3,783,853	3.78	0.003
H1_Gt14(31)_D	12,712	0.013	3,654,038	3.65	0.003
H2_Gt14(31)_D	12,712	0.013	3,650,216	3.65	0.003
Lpn-Corby	8,569	0.009	3,576,470	3.58	0.009
L06-153_Cl_Gt71(135)_D	11,960	0.012	3,405,980	3.41	0.012
L06-129_Cl_Gt71(135)_D	11,957	0.012	3,406,112	3.41	0.012
A166_Gt8(142)_Ps	13,650	0.014	3,353,128	3.35	0.014
L04-041_Cl_Gt30(137)_D	14,500	0.015	3,522,671	3.52	0.015
L05-341_Cl_Gt8(132)_D	13,146	0.013	3,410,856	3.41	0.013

Table S5: Specific genes for each *L. pneumophila* isolate (hypothetical proteins were excluded)

Isolate desgnation	Gene No.	Protein function
L09-313_Cl_Gt84(116)_D	O6C93_01440	TolC family protein
L01-443_Cl_Gt64(74)_D	O6D81_11770	IS6 family transposase
	O6D81_12360	PHA synthase
	O6D81_12420	c-type cytochrome
	O6D81_12425	c-type cytochrome
	O6D81_12430	c-type cytochrome
	O6D81_12440	TerC family protein
	**O6D81_12455**	**reverse transcriptase domain-containing protein**
	**O6D81_12640**	**MBL fold metallo-hydrolase**
	O6D81_12660	heavy metal-binding domain-containing protein
	O6D81_12690	TolC family protein
	O6D81_12705	efflux RND transporter permease subunit
	**O6D81_12815**	**MBL fold metallo-hydrolase**
	O6D81_12840	GGDEF domain-containing protein
	O6D81_12860	TolC family protein
	O6D81_12870	efflux RND transporter permease subunit
	O6D81_12935	efflux RND transporter permease subunit
	O6D81_12945	efflux RND transporter permease subunit
	O6D81_12955	TolC family protein
	O6D81_12520	2Fe-2S iron-sulfur cluster-binding protein
	O6D81_12955	TolC family protein
	O6D81_15015	efflux RND transporter permease subunit
A129_Gt64(74)_Ps	**O6D13_00025**	**Antitoxin igA-2**
	O6D13_00035	Tyrosine recombinase XerC
	O6D13_00040	ORF6N domain protein
	O6D13_00085	GGDEF domain-containing protein
	O6D13_00090	Tn3 family transposase
	O6D13_00095	recombinase family protein
	O6D13_00100	alpha/beta hydrolase
	O6D13_00115	response regulator
	O6D13_00125	zinc ribbon domain-containing protein
	O6D13_00140	efflux RND transporter periplasmic adaptor subunit

	**O6D13_10510**	**TolC family protein**
	**O6D13_00230**	**ParB/RepB/Spo0J family partition protein**
	O6D13_00290	carbon storage regulator CsrA
	O6D13_00355	TIGR03752 family integrating conjugative element protein
	**O6D13_00365**	**conjugative transfer ATPase**
	**O6D13_00375**	**TIGR03756 family integrating conjugative element protein**
	O6D13_00380	integrating conjugative element protein
	O6D13_00385	conjugal transfer protein TraG N-terminal domain-containing protein
	O6D13_00410	DNA topoisomerase III
	O6D13_00470	integrase domain-containing protein
	O6D13_03035	integrase domain-containing protein
	**O6D13_03065**	**ATP-binding cassette domain-containing protein**
	O6D13_03090	efflux RND transporter periplasmic adaptor subunit
	O6D13_03095	efflux RND transporter permease subunit
	O6D13_03150	tyrosine-type recombinase/integrase
	O6D13_03225	DUF4156 domain-containing protein
	O6D13_13310	single-stranded DNA-binding protein
	**O6D13_13975**	**lpg2844 family Dot/Icm T4SS effector**
	O6D13_14710	IS21 family transposase
H29_Gt22(100)_D	O6D14_10380	HipA N-terminal domain-containing protein
H39_Gt4(17)_D	O6D15_00630	ACR3 family arsenite efflux transporter
	O6D15_00635	arsenate reductase ArsC
	**O6D15_00640**	**ArsI/CadI family heavy metal resistance metalloenzyme**
	**O6D15_00645**	**metalloregulator ArsR/SmtB family transcription factor**
	O6D15_06785	Tn3 family transposase
A139_Gt4(17)_Ps	**O6D12_04050**	**cell division protein FtsI/penicillin binding protein 2**
A5_Gt4(17)_Ps	**O6C87_09020**	**SdhB protein, substrate of the Dot/Icm system**
A29_Gt6(18)_Ps	O6D23_02780	cation diffusion facilitator family transporter
	**O6D23_02795**	**type II toxin-antitoxin system HicB family antitoxin**
	O6D23_02850	patatin-like phospholipase family protein
	O6D23_02855	DUF3734 domain-containing protein
	**O6D23_02870**	**helix-turn-helix transcriptional regulator**
	O6D23_02910	ATP-binding protein
	**O6D23_02915**	**ParA family protein**
	O6D23_02945	TraY domain-containing protein
	O6D23_05230	MFS transporter
L09-329_Cl_Gt75(49)_D	O6C51_05560	HNH endonuclease signature motif containing protein

	**O6C51_05570**	**helix-turn-helix transcriptional regulator**
	O6C51_14225	BREX-1 system adenine-specific DNA-methyltransferase PglX
	O6C51_14230	BREX-1 system phosphatase PglZ type A
L11-209_Cl_Gt29(27)_D	**O6D35_07945**	**DNA repair exonuclease**
	O6D35_07950	AAA family ATPase
	O6D35_10455	septation initiation protein
L02-521_Cl_Gt29(27)_D	**L02_521_01364**	**type IV pilus biogenesis protein PilQ**
	**O6D27_09325**	**pilus assembly protein FimV**
	O6D27_13310	T4SS effector NAD-dependent ubiquitin ligase SdeA
	**L02_521_02661**	**SidE phosphodiesterase domain-containing protein**
L12-317_Cl_Gt72(66)_D	O6D34_00100	ATP-binding protein
	O6D34_07330	helicase-related protein
	O6D34_07345	DEAD/DEAH box helicase family protein
L10-091_Cl_Gt69(69)_D	O6E59_01085	macro domain-containing protein
	O6E59_10395	GNAT family N-acetyltransferase
	O6E59_10490	diguanylate cyclase
	O6E59_10495	CZB domain-containing protein
	O6E59_10500	GGDEF domain-containing protein
	**O6E59_10515**	**class I SAM-dependent methyltransferase**
	O6E59_10550	FIST C-terminal domain-containing protein
	**O6E59_10555**	**DHA2 family efflux MFS transporter permease subunit**
	**O6E59_10560**	**HlyD family secretion protein**
	**O6E59_10565**	**MarR family transcriptional regulator**
	**O6E59_10570**	**efflux transporter outer membrane subunit**
	O6E59_10575	sodium:calcium antiporter
	O6E59_10585	HlyD family efflux transporter periplasmic adaptor subunit
	O6E59_10590	ribosome-associated ATPase/putative transporter RbbA
	O6E59_10595	ABC transporter permease
	**O6E59_10625**	**Hsp20/alpha crystallin family protein**
	O6E59_10645	class I SAM-dependent methyltransferase
	**O6E59_10650**	**type II toxin-antitoxin system PemK/MazF family toxin**
	O6E59_13710	GNAT family N-acetyltransferase
L10-023_Cl_Gt75(49)_D	**Lpn-L10-023_00139**	**SdhB protein, substrate of the Dot/Icm system**
	Lpn-L10-023_00204	N-6 DNA Methylase

Lpn-L10-023_00228	Inner membrane protein YedI
**Lpn-L10-023_00286**	**heme oxygenase**
**Lpn-L10-023_00290**	**heavy metal transporting P-type ATPase, cation transporting**
Lpn-L10-023_00336	nicotinate phosphoribosyltransferase
Lpn-L10-023_00381	translation elongation factor Tu (EF-Tu)
**Lpn-L10-023_00444**	**SdhA, substrate of the Dot/Icm system**
Lpn-L10-023_00468	signal recognition particle protein Ffh
Lpn-L10-023_00607	dihydrolipoamide succinyltransferase
Lpn-L10-023_00761	DNA topoisomerase IV subunit B
Lpn-L10-023_00967	A/G specific adenine glycosylase
**Lpn-L10-023_00995**	**penicillin binding protein 1A**
Lpn-L10-023_01186	DNA integration/recombination/inversion protein
**Lpn-L10-023_01251**	**permeases of drug/transporter**
Lpn-L10-023_01275	2-hydroxy-6-oxo-6-phenylhexa-2,4-dienoate hydrolase
Lpn-L10-023_01447	thymidylate kinase
Lpn-L10-023_01448	thymidylate kinase
**Lpn-L10-023_01576**	**(type IV) pilus assembly protein PilC**
**Lpn-L10-023_01578**	**type 4 (IV) prepilin-like protein leader peptide processing enzyme PilD**
Lpn-L10-023_01590	glutamate-1-semialdehyde-2,1-aminomutase
Lpn-L10-023_01637	TolA colicin import membrane protein
Lpn-L10-023_02146	transporter, Zip family
Lpn-L10-023_02235	membrane protein
Lpn-L10-023_02328	sensory box histidine kinase/response regulator
Lpn-L10-023_02334	polyketide synthase, type I
Lpn-L10-023_02335	polyketide synthase, type I
Lpn-L10-023_02336	polyketide synthase, type I
Lpn-L10-023_02370	purine NTPase, putative
Lpn-L10-023_02384	3-oxoacyl-(acyl carrier protein) synthase III
Lpn-L10-023_02501	hypothetical protein
**Lpn-L10-023_02530**	**Legionella vir region protein**
Lpn-L10-023_02531	global regulator (carbon storage regulator)
Lpn-L10-023_02560	cellulose synthase subunit BcsC
Lpn-L10-023_02566	Na(+)/H(+) antiporter 1 (Sodium/proton antiporter 1)
Lpn-L10-023_02568	integral membrane protein
Lpn-L10-023_02572	OsmC-like protein
Lpn-L10-023_02574	potassium efflux system KefA
Lpn-L10-023_02575	copper efflux ATPase
Lpn-L10-023_02657	Na(+)/H(+) antiporter 1 (Sodium/proton antiporter 1
Lpn-L10-023_02720	hypothetical, patatin-like phospholipase
Lpn-L10-023_02756	glutathione S-transferase

	Lpn-L10-023_02780	hydrogenase expression/formation protein
	Lpn-L10-023_02781	hydrogenase expression/formation protein
	**Lpn-L10-023_02834**	**outer membrane efflux protein (RND multidrug efflux)**
	Lpn-L10-023_02847	hypothetical protein with coiled coil domain protein
	Lpn-L10-023_02848	hypothetical protein with coiled coil domain protein
	Lpn-L10-023_03007	phospho-N-acetylmuramoyl- pentapeptidetransferase
	Lpn-L10-023_03012	chromosome segregation SMC protein
	Lpn-L10-023_03084	cation transporting ATPase PacS
	Lpn-L10-023_03135	uroporphyrinogen III methylase
	Lpn-L10-023_03137	protoporphyrinogen IX and coproporphyrinogen III oxidase HemY
	Lpn-L10-023_03338	outer membrane efflux protein
	Lpn-L10-023_03386	peroxynitrite reductase, AhpC/Tsa family
	Lpn-L10-023_03416	tetrapyrrole (corrin/porphyrin) methylase
	Lpn-L10-023_03423	GTP binding protein in thiophene and furan oxidation (GTPase)
H23_Gt14(30)_D	**O6C86_14460**	**efflux RND transporter permease subunit**
	**O6C86_14460**	**efflux RND transporter permease subunit**
	O6C86_10150	conjugal transfer protein TraH
	**O6C86_09200**	**TraU family protein**
	**O6C86_09230**	**TraE/TraK family type IV conjugative transfer system protein**
	**O6C86_09235**	**TraE/TraK family type IV conjugative transfer system protein**
	O6C86_10150	conjugal transfer protein TraH
	O6C86_17105	LuxR C-terminal-related transcriptional regulator
	O6C86_17105	LuxR C-terminal-related transcriptional regulator
	H23_02368	lipolytic enzyme
	O6C86_17085	DNA adenine methylase
	O6C86_17090	NADPH-dependent 7-cyano-7-deazaguanine reductase QueF
H3_Gt14(31)_D	**LpnH3D14_00327**	**SdbA protein, putative substrate of the Dot/Icm system**
	LpnH3D14_01633	TnpA transposase
H1_Gt14(31)_D	O6C83_15670	integrase domain-containing protein
	O6C83_15670	integrase domain-containing protein
L04-041_Cl_Gt30(137)_D	**L04_041_00187**	**cadmium translocating P-type ATPase CadA**
	O6C82_01235	conjugative coupling factor TraD, PFGI-1 class
	L04_041_00251	conjugative coupling factor TraD
	O6C82_01990	ABC transporter permease
	O6C82_02290	AAA family ATPase
	O6C82_03960	(outer) membrane protein
	O6C82_13255	GIY-YIG nuclease superfamily protein

	L04_041_02904	SdcA
	**L04_041_02906**	**SidC protein (substrate of the Dot/Icm system)**
L02-465_Cl_Gt27(133)_D	O6D28_09245	ATP-binding protein
	O6D28_09275	MFS transporter
	O6D28_09280	peptide deformylase
	O6D28_14745	alpha/beta fold hydrolase
	O6D28_10135	FAD-binding oxidoreductase
	O6D28_10265	GGDEF domain-containing protein
	O6D28_10270	diguanylate cyclase
	O6D28_10315	cation-translocating P-type ATPase
	O6D28_05830	ISAs1 family transposase
	O6D28_10095	F0F1 ATP synthase subunit beta
	O6D28_14780	F0F1 ATP synthase subunit A
	O6D28_14785	F0F1 ATP synthase subunit A
A194_Gt40(47)_Ps	LpnA194_02940	hypothetical histidine-rich protein
A193_Gt40(47)_Ps	O6C92_00445	translocation/assembly module TamB domain-containing protein
	O6C92_09225	amino acid dehydrogenase
	A193_02478	Primosomal protein N' (replication factor Y)
	O6C92_15530	HslU--HslV peptidase ATPase subunit
L05-341_Cl_Gt8(132)_D	O6C52_01120	AAA family ATPase
	O6C52_06635	cyclic nucleotide-binding domain-containing protein
	**O6C52_11395**	**Vir protein**
	**L05_341_02307**	**type I restriction enzyme EcoKI subunit R**
	**L05_341_02308**	**Plasmid maintenance system killer protein**
	O6C52_11530	helix-turn-helix domain-containing protein
	O6C52_11540	nucleotide-binding protein
L06-153_Cl_Gt71(135)_D	O6D25_07255	tetratricopeptide repeat protein
A138_Gt9(92)_Ps	**A138_02923**	**Multifunctional virulence effector protein DrrA**
A127_Gt10(93)_Ps	O6D26_08745	NAD-dependent formate dehydrogenase
	O6D26_08750	NAD-dependent formate dehydrogenase
	A127_02037	sensory box protein, EAL domain, GGDEF domain, signal transduction protein
A166_Gt8(142)_Ps	**O6C96_02340**	**IcmE (DotG)**
	**O6C96_02805**	**cyclic nucleotide-binding domain-containing protein**
	O6C96_10115	serine/threonine protein kinase

	O6C96_10125	serine/threonine-protein kinase
	O6C96_13195	hypothetical histidine-rich protein
L04-545_Cl_Gt40(47)_D	O6D22_05960	Ankyrin repeats
	O6D22_07115	septation initiation protein

**Table S6**: **Nucleotide Polymorphisms in cluster six of L.** ***pneumophila*** **isolates H1, H2, and H23 from the HZI DWSS relative to the H3 PacBio reference sequence obtained.**							
**Nucleotide Polymorphism**	**Label (locus tag) ref genome**	***L. pneumophila*** **Isolate**	**Gene Name**	**Gene Product**	**Gene Function**	**Length (bp)**	**Virulence Similarity (%)**
A → T	LpnH3D14_00006	H2	*ppeA*	Biosynthetic arginine de-carboxylase	Intracellular Growth	1,891	NV
C → T	LpnH3D14_00027	H23	*yhdG_1*	Amino acid transporter	Survival *in vivo*	1,391	NV
C → T	LpnH3D14_00935	H1, H2, H23	*bepC*	Outer membrane efflux protein	Survive inside the host cells, restores drug efflux	1,724	NV
T → G	LpnH3D14_00978	H1	*mmgC*	Acyl-CoA dehydrogenase		1,154	NV
A → G	LpnH3D14_01656	H23		Alpha/beta hydrolase		983	NV
C → T	LpnH3D14_02694	H1, H2, H23	*ankF*	Dot/Icm type IV secretion system effector (Ankyrin repeats)	Host microbe interaction, Intracellular replication	2,765	96
G → T	LpnH3D14_02706	H23		Aspartyl/Asparaginyl beta hydrolase		719	NV
C → T	LpnH3D14_02929	H1, H2, H23	dotA	Phagosome trafficking protein DotA	Bacterial replication, virulence, Evasion of Endocytic pathway	3,119	79

C → T	LpnH3D14_03123	H23		Hypothetical protein		1,289	NV

NV: Non-Virulence Gene

Table S7: Gene content according to MAUVE aligner in the studied areas. Hypothetical proteins were excluded			
**Locus Tag**	**Gene Name**	**Product**	**Length (bp)**
**Area 1**			
LpnA194_02079		Acetyltransferase (GNAT) family protein	437
LpnA194_02080	dpnM	Modification methylase DpnIIA	1,412
LpnA194_02081	dlpA_1	DlpA protein	719
LpnA194_02082		Phosphotransferase enzyme family protein	968
LpnA194_02083		aminoglycoside phosphotransferase	1,436
LpnA194_02085		shikimate kinase	779
LpnA194_02086		Enterochelin esterase	1,415
LpnA194_02088	abcT3_4	ABC transporter, ATP binding protein	1,547
LpnA194_02090		transcriptional regulator, MerR family, mercury resistance	944
LpnA194_02091		beta-lactamase AmpS	800
LpnA194_02092	ftsI4_2	cell division protein FtsI/penicillin binding protein 2	1,922
LpnA194_02093	mecI_2	Methicillin resistance regulatory protein mecI	422
LpnA194_02094		transcriptional regulator SkgA, mercury resistance	749
LpnA194_02097		acetyltransferase, GNAT family	602
LpnA194_02098		reverse transcriptase	1,556
LpnA194_02100		acetyltransferase, GNAT family	482
LpnA194_02101	grpB_2	glutamate rich protein GrpB	956
LpnA194_02102		nucleotidyltransferase PLUS glutamate rich protein GrpB PLUS ribosomal protein alanine acetyltransferase	794
LpnA194_02104		pyridoxamine 5'-phosphate oxidase	581
LpnA194_02105		phage repressor	622
LpnA194_02106	lvrA_2	Legionella vir region protein	872
LpnA194_02107	lvrC	Legionella vir region protein	197
LpnA194_02108		putative exported protein	434
LpnA194_02121		Type IV secretory protein VirB4 component	2,774
LpnA194_02122		TraU protein	995
LpnA194_02123		membrane protein, Tfp pilus assembly, pilus retraction ATPase PilT	1,382
LpnA194_02127		conjugative coupling factor TraD	2,006
LpnA194_02129		Putative HTH-type transcriptional regulator/MT0914	572

LpnA194_02130		Avidin family protein	413
LpnA194_02132		putative secreted esterase	1,556
LpnA194_02133		Bacterial regulatory proteins, luxR family	800
LpnA194_02134	murE3_1	UDP-N-acetylmuramyl tripeptide synthase	1,226
LpnA194_02137	aph_2	spectinomycin phosphotransferase	995
LpnA194_02138		glyoxylase domain hypothetical protein	365
LpnA194_02139		short chain dehydrogenase	734
LpnA194_02140	yafP	putative N-acetyltransferase YafP	1,697
LpnH3D14_02136	fni	isopentenyl-diphosphate delta-isomerase	1,028
LpnH3D14_02137	mvaA	hydroxymethylglutaryl CoA reductase	1,298
LpnH3D14_02139		tRNA-Lys	75
LpnH3D14_02140		tRNA-Lys	75
LpnH3D14_02141		tRNA-Arg	76
LpnH3D14_02142		site specific recombinase	1,160
LpnH3D14_02143	nucS	Endonuclease NucS	1,004
LpnH3D14_02144		Acetyltransferase (GNAT) family protein	485
LpnH3D14_02145		proline/betaine transport protein like protein	1,274
LpnH3D14_02146		putative acetyltransferase	512
LpnH3D14_02147		lipolytic enzyme	686
LpnH3D14_02148		transcription regulator protein, response regulator containing CheY-like receiver domain and HTH DNA-binding domain	803
LpnH3D14_02150	tylM1	dTDP-3-amino-3,6-dideoxy-alpha-D-glucopyranose N,N-dimethyltransferase	824
LpnH3D14_02152		topology modulation protein	542
LpnH3D14_02154		aminoglycoside 6'-N-acetyltransferase	545
LpnH3D14_02155		acetyltransferase, GNAT family	554
LpnH3D14_02156		transcriptional regulator, MerR family, mercury resistance	1,040
LpnH3D14_02157		methyltransferase	881
LpnH3D14_02158		Acetyltransferase (GNAT) family protein	419
LpnH3D14_02159		recombination protein F	1,160
LpnH3D14_02160		Cupin domain protein	386
LpnH3D14_02162		O-antigen acetylase	1,148
LpnH3D14_02163		serine/threonine protein kinase	1,001
LpnH3D14_02164	phaB_4	acetyoacetyl CoA reductase	782
LpnH3D14_02165		sepiapterin reductase	794
LpnH3D14_02165	dpnM	Modification methylase DpnIIA	1,412
LpnH3D14_02168	dlpA_1	DlpA protein	716
LpnH3D14_02169		Phosphotransferase enzyme family protein	968

LpnH3D14_02170		aminoglycoside phosphotransferase	1,436
LpnH3D14_02172	aroK_2	shikimate kinase	779
LpnH3D14_02173	mdtK	Multidrug resistance protein MdtK	1,364
LpnH3D14_02174		transcriptional regulator, LysR family	863
LpnH3D14_02175		major facilitator superfamily transporter	1,175
LpnH3D14_02176		Peptidase family S41	1,337
LpnH3D14_02177		phage repressor	662
LpnH3D14_02178	lvrA_2	Legionella vir region protein	872
LpnH3D14_02179	lvrC_2	Legionella vir region protein	197
LpnH3D14_02180		putative exported protein	434
LpnH3D14_02193		Type IV secretory protein VirB4 component	2,774
LpnH3D14_02194		TraU protein	977
LpnH3D14_02195		membrane protein, Tfp pilus assembly, pilus retraction ATPase PilT	1,382
LpnH3D14_02196		membrane protein	1,568

Table S7: Gene content according to MAUVE aligner in the studied areas. Hypothetical proteins were excluded (Area 2)

**Locus Tag**	**Gene Name**	**Product**	**Length (bp)**
corby_02494		lipolytic enzyme	683
corby_02495		transcription regulator protein, response regulator containing CheY-like receiver domain and HTH DNA-binding domain	803
Corby_02499	cadA_2	cadmium translocating P-type ATPase CadA	503
Corby_02500		cadmium efflux ATPase	1,433
Corby_02502	cadA_3	cadmium translocating P-type ATPase CadA	2,135
Corby_02503	helA_2	cobalt/zinc/cadmium efflux RND transporter, permease protein HelA	3,149
Corby_02504	helB_2	cation efflux system HelB	1,256
Corby_02505	helC_2	cobalt/zinc/cadmium efflux RND transporter, outer membrane protein	1,244
Corby_02506		reverse transcriptase	1,364
Corby_02511		reverse transcriptase	1,199
Corby_02512		transposase IS4	1,460
Corby_02513		phage repressor	662
Corby_02514	lvrA_2	Legionella vir region protein	872
Corby_02515	lvrB_2	Legionella vir region protein	383
Corby_02516	lvrC	Legionella vir region protein	197
Corby_02517		putative exported protein	434
Corby_02528		exported membrane protein	1,313
Corby_02530		Type IV secretory protein VirB4 component	2,774

Corby_02531		TraU protein	995
Corby_02532		membrane protein, Tfp pilus assembly, pilus retraction ATPase PilT	1,382
Corby_02533		membrane protein	1,568
Corby_02536		conjugative coupling factor TraD	2,006

**Table S8: Genes of interest in genomic islands of *L. pneumophila* isolates as identified by IslandViewer4**					
Branch	Isolate	Gene	Locus tag	Gene Length (bp)	Product
3 HL 06041085	H34		H34_02359	965	major outer membrane protein
		tufA_2	H34_02301	917	translation elongation factor Tu (EF-Tu)
			H34_02302	341	preprotein translocase subunit SecE
		nusG	H34_02303	548	transcription antitermination protein NusG
		rplK	H34_02304	434	50S ribosomal protein L11
		rplA	H34_02305	695	50S ribosomal protein L1
		rplJ	H34_02306	533	50S ribosomal protein L10
		rplL	H34_02307	380	50S ribosomal protein L7/L12
		rpoB	H34_02308	4,106	DNA-directed RNA polymerase beta subunit
		rpoC	H34_02309	4,247	DNA-directed RNA polymerase subunit beta'
		rpsL	H34_02310	380	30S ribosomal protein S12
		rpsG	H34_02311	527	30S ribosomal protein S7
		fusA	H34_02312	2,084	translation elongation factor G (EF-G)
	H35	*ccmC*	H35_00671	755	heme exporter protein CcmC
		ccmD	H35_00670	158	heme exporter protein CcmD
		ccmE	H35_00669	431	cytochrome c-type biogenesis protein CcmE
		ccmF	H35_00668	1,952	cytochrome c-type biogenesis protein CcmF
		ccmG	H35_00667	533	cytochrome C biogenesis protein
		ccmH	H35_00666	401	c-type cytochrome biogenesis protein CcmH
		cycH	H35_00665	686	cytochrome c type biogenesis protein CcmH
4 Paris	A29		A29_00568	206	cold shock domain family protein
	A5		A5_02183	986	Microvirus H protein (pilot protein)
			A5_02184	1,541	Bacteriophage replication gene A protein (GPA)
			A5_02185	260	Phage protein C
			A5_02186	458	Bacteriophage scaffolding protein D
			A5_02187	116	Microvirus J protein
	A139		A139_02127	1,025	Bacteriophage replication gene A protein (GPA)
			A139_02128	260	Phage protein C
			A139_02129	458	Bacteriophage scaffolding protein D
			A139_02130	116	Microvirus J protein
			A139_02131	1,283	Capsid protein (F protein)
			A139_02132	527	Major spike protein (G protein)
			A139_02133	986	Microvirus H protein (pilot protein)

5 Pontiac	L10-091	*hspC2_1*	L10-091_02122	491	small heat shock protein HspC2
			L10-091_02123	587	heat shock hsp20
6 Alcoy	L02-465		L02-465_00734	425	hemin binding protein Hbp
	A194	*cya*	A194_00644	15,197	Bifunctional hemolysin/adenylate cyclase precursor
	A112	*chuR*	A112_02425	1,475	Anaerobic sulfatase-maturating enzyme
	H23	*ampG*	H23_01834	1,247	beta lactamase induction signal transducer AmpG
			H23_02025	1,361	metallo-beta lactamase family protein
			H23_01996	1,424	Bacteriophage replication gene A protein (GPA)
			H23_01997	260	Phage protein C
			H23_01998	458	Bacteriophage scaffolding protein D
			H23_01999	116	Microvirus J protein
			H23_02000	1,283	Capsid protein (F protein)
			H23_02001	527	Major spike protein (G protein)
			H23_02002	986	Microvirus H protein (pilot protein)
	H1	*ampG*	H1_02766	1,247	beta lactamase induction signal transducer AmpG
			H1_00952	1,361	metallo-beta lactamase family protein
			H1_02730	416	Bacteriophage scaffolding protein D
			H1_02731	116	Microvirus J protein
			H1_02732	1,283	Capsid protein (F protein)
			H1_02733	527	Major spike protein (G protein)
			H1_02734	986	Microvirus H protein (pilot protein)
			H1_02735	1,541	Bacteriophage replication gene A protein (GPA)
	H2		H2_02312	1,361	metallo-beta lactamase family protein
	H3		H3_03288	1,361	metallo-beta lactamase family protein
	L06-129	*htpX_3*	L06-129_01854	965	heat shock protein, protease HtpX
			L06-129_01853	470	Phosphate-starvation-inducible E
		trxA3_2	L06-129_01852	440	thioredoxin
			L06-129_01848	569	small HspC2 heat shock protein
		cas1	L06-129_01845	968	CRISPR-associated endonuclease Cas1
		cas3	L06-129_01844	3,395	CRISPR-associated nuclease/helicase Cas3 subtype I-F/YPEST
		csy1	L06-129_01843	1,265	CRISPR-associated protein Csy1
		htpX_3	L06-129_01854	965	heat shock protein, protease HtpX
			L06-129_01853	470	Phosphate-starvation-inducible E
			L06-129_01848	569	small HspC2 heat shock protein

Table S9. Number and distribution of the 8 most frequently identified eukaryotic motifs and heat shock proteins (HSP) within the 55 *L. pneumophila* strains studied. Numbers represent the number of proteins containing the respective eukaryotic motif.

Strain	Ankyrin repeats	F-box	U-box	P-Kinase	LRR	P450	SET	HAD	HSP
A129_Gt64(74)_Ps	12	3	1	6	6	0	1	1	16
A156_Gt64(74)_Ps	12	3	1	6	6	0	1	1	15
L01-443_Cl_Gt64(74)_D	12	3	1	7	7	1	1	1	15
L09-313_Cl_Gt84(116)_D	12	3	1	6	6	0	1	1	16
Lpn-LPE509	12	3	1	7	6	0	1	1	15
Lpn-lpm7613	11	3	1	7	6	0	1	1	16
Lpn-ATCC43290	12	3	1	6	6	0	1	1	16
Lpn-Philadelphia1	12	3	1	7	6	0	1	1	15
Lpn-Thunderbay	12	3	1	6	6	0	1	1	16
A15_Gt12(84)_Ps	16	1	1	11	7	1	1	1	13
Lpn-Lens	15	1	0	7	5	1	1	1	14
Lpn-Lorraine	14	2	1	9	7	1	1	1	13
H29_Gt22(100)_D	12	2	1	8	3	1	1	1	14
H34_Gt22(102)_D	12	2	1	8	3	1	1	1	14
H35_Gt22(102)_D	12	2	1	8	3	1	1	1	14
Lpn-D7630	12	2	1	8	3	1	1	1	14
Lpn-D7631	12	2	1	8	3	1	1	1	14
Lpn-D7632	12	2	1	8	3	1	1	1	14
Lpn-HL06041035	11	1	1	7	5	1	1	1	15
A5_Gt4(17)_Ps	15	2	1	12	4	1	1	1	13
A29_Gt6(18)_Ps	17	2	1	10	4	1	1	1	13
A131_Gt6(18)_Ps	17	2	1	10	4	1	1	1	13
A139_Gt4(17)_Ps	15	1	1	10	4	1	1	1	13
H39_Gt4(17)_D	17	2	1	9	4	1	1	1	13
Lpn-OLDA	16	2	1	11	4	1	1	1	13
Lpn-Paris	16	2	1	11	4	1	1	1	13
L02-521_Cl_Gt29(27)_D	13	2	1	11	3	1	1	1	13
L09-329_Cl_Gt75(49)_D	13	1	1	11	3	1	1	1	13
L10-091_Cl_Gt69(69)_D	12	2	1	13	3	1	1	1	15
L11-209_Cl_Gt29(27)_D	12	1	1	11	3	1	1	1	13
L12-317_Cl_Gt72(66)_D	11	2	1	12	3	1	1	1	13
L10-023_Cl_Gt75(49)_D	11	1	1	11	3	1	1	1	14
Lpn-Toronto	12	2	0	10	3	1	1	1	14
Lpn-Pontiac	13	3	1	11	3	1	1	1	13
A108_Gt10(93)_Ps	12	1	0	6	3	1	1	1	15

A112_Gt10(93)_Ps	12	1	0	6	3	1	1	1	16
A114_Gt10(93)_Ps	12	1	0	6	3	1	1	1	16
A127_Gt10(93)_Ps	12	1	0	6	3	1	1	1	16
A138_Gt9(92)_Ps	12	1	0	6	3	1	1	1	16
A166_Gt8(142)_Ps	13	1	0	7	3	1	1	1	15
A193_Gt40(47)_Ps	16	1	0	7	3	1	1	1	14
A194_Gt40(47)_Ps	16	1	0	7	3	1	1	1	15
A195_Gt40(47)_Ps	16	1	0	7	3	1	1	1	15
Lpn-Alcoy 2300/99	14	1	0	7	3	1	1	1	15
Lpn-Corby	14	1	0	8	3	1	1	1	15
H1_Gt14(31)_D	14	1	0	8	2	1	1	1	16
H2_Gt14(31)_D	14	1	0	8	2	1	1	1	16
H3_Gt14(31)_D	14	1	0	8	2	1	1	1	16
H23_Gt14(30)_D	15	1	0	10	2	1	1	1	16
L02-465_Cl_Gt27(133)_D	11	1	0	8	5	1	1	1	14
L04-041_Cl_Gt30(137)_D	11	1	0	6	3	1	1	1	15
L04-545_Cl_Gt40(47)_D	16	1	0	6	3	1	1	1	16
L05-341_Cl_Gt8(132)_D	12	1	0	6	3	1	1	1	15
L06-129_Cl_Gt71(135)_D	12	1	0	7	3	1	1	1	17
L06-153_Cl_Gt71(135)_D	12	1	0	7	3	1	1	1	17

Gt, MLVA genotype; _Ps, West Bank; _D, Germany, ANK, ankyrin; F-box, F-box domain; LLR, leucine rich repeats; P450, cytochrome_P450; P-kinases (protein kinases); U-box, U-box domain; HAD, HAD-superfamily hydrolase; SET, SET domain; and HPS, Heat Shock proteins.

Table S10 : Nucleotide identity values of orthologous pore-forming activity genes with respect to the BLASTp search against the VFDB using *L. pneumophila* strain Philadelphia1 as default reference genome

***L. pneumophila* strain**	***Secretion system***											***Toxin***
	**Dot/Icm type IVB secretion system**											**RtxA**
	icmT	***icmS***	***icmR***	***icmQ***	***icmL/dotI***	***icmK/dotH***	***icmE/dotG***	***icmC/dotE***	***dotB***	***dotA***	***icmW***	***rtxA***
	**Id***											
**Lpn-Corby**	82%	100%	95%	100%	84%	84%	88%	91%	100%	85%	98%	85%
**Lpn-Alcoy**	82%	100%	95%	100%	84%	84%	88%	99%	100%	81%	98%	85%
**L02-465_Cl_Gt27(133)_D**	82%	100%	95%	100%	84%	84%	88%	99%	100%	81%	99%	85%
**L04-545_Cl_Gt40(47)_D**	82%	100%	95%	100%	84%	84%	88%	99%	100%	81%	98%	84%
**A193_Gt40(47)_Ps**	82%	100%	95%	100%	84%	84%	88%	99%	100%	78%	98%	84%
**A194_Gt40(47)_Ps**	82%	100%	95%	100%	84%	84%	88%	99%	100%	78%	98%	84%
**A195_Gt40(47)_Ps**	82%	100%	95%	100%	84%	84%	88%	99%	100%	78%	98%	84%
**A138_Gt9(92)_Ps**	82%	100%	95%	100%	84%	83%	88%	99%	100%	78%	98%	75%
**A112_Gt10(93)_Ps**	82%	100%	95%	100%	84%	83%	88%	99%	100%	78%	98%	75%
**A114_Gt10(93)_Ps**	82%	100%	95%	100%	84%	83%	88%	99%	100%	78%	98%	75%
**A127_Gt10(93)_Ps**	82%	100%	95%	100%	84%	83%	88%	99%	100%	78%	98%	75%
**A108_Gt10(93)_Ps**	82%	100%	95%	100%	84%	83%	88%	99%	100%	78%	98%	75%
**H3_Gt14(31)_D**	82%	100%	95%	100%	84%	83%	87%	99%	100%	79%	98%	86%
**H23_Gt14(30)_D**	82%	100%	95%	100%	84%	83%	87%	99%	100%	78%	98%	86%
**H1_Gt14(31)_D**	82%	100%	95%	100%	84%	83%	87%	99%	100%	78%	98%	86%
**H2_Gt14(31)_D**	82%	100%	95%	100%	84%	83%	87%	99%	100%	78%	98%	86%
**L06-153_Cl_Gt71(135)_D**	82%	100%	95%	100%	84%	84%	88%	99%	100%	80%	98%	85%
**L06-129_Cl_Gt71(135)_D**	82%	100%	95%	100%	84%	84%	88%	99%	100%	80%	98%	85%

**A166_Gt8(142)_Ps**	82%	100%	95%	100%	84%	84%	89%	99%	100%	80%	98%	84%
**L04-041_Cl_Gt30(137)_D**	82%	100%	95%	100%	84%	84%	88%	99%	100%	80%	98%	85%
**L05-341_Cl_Gt8(132)_D**	82%	100%	95%	100%	84%	84%	88%	99%	100%	80%	98%	84%
**Lpn-Pontiac**	82%	100%	96%	99%	84%	82%	88%	100%	100%	88%	99%	80%
**L10-091_Cl_Gt69(69)_D**	82%	100%	96%	99%	84%	82%	88%	100%	100%	81%	99%	75%
**L12-317_Cl_Gt72(66)_D**	82%	100%	96%	99%	84%	82%	88%	100%	100%	81%	99%	75%
**L11-209_Cl_Gt29(27)_D**	82%	100%	96%	99%	84%	82%	88%	100%	100%	81%	99%	80%
**L10-023_Cl_Gt75(49)_D**	82%	100%	96%	99%	84%	82%	88%	100%	100%	81%	99%	80%
**L09-329_Cl_Gt75(49)_D**	82%	100%	96%	99%	84%	82%	88%	100%	100%	81%	99%	80%
**L02-521_Cl_Gt29(27)_D**	82%	100%	96%	99%	84%	82%	88%	100%	100%	81%	99%	80%
**Lpn-Paris**	82%	100%	96%	99%	84%	82%	89%	100%	99%	81%	99%	83%
**H39_Gt4(17)_D**	82%	100%	96%	99%	84%	82%	89%	100%	100%	81%	99%	83%
**A139_Gt4(17)_Ps**	82%	100%	96%	99%	84%	82%	89%	100%	99%	81%	99%	83%
**A5_Gt4(17)_Ps**	82%	100%	96%	99%	84%	82%	89%	100%	99%	81%	99%	83%
**A29_Gt6(18)_Ps**	82%	100%	96%	99%	84%	82%	89%	100%	99%	81%	99%	83%
**A131_Gt6(18)_Ps**	82%	100%	96%	99%	84%	82%	89%	100%	99%	81%	99%	83%
**Lpn-HL06041035**	82%	99%	97%	99%	84%	82%	87%	99%	100%	81%	99%	85%
**H34_Gt22(102)_D**	82%	99%	97%	99%	84%	82%	87%	99%	100%	81%	99%	85%
**H29_Gt22(100)_D**	82%	99%	97%	99%	84%	82%	87%	99%	100%	81%	99%	85%
**H35_Gt22(102)_D**	82%	99%	97%	99%	84%	82%	87%	99%	100%	81%	99%	85%
**Lpn-Lorraine**	82%	100%	97%	100%	84%	82%	87%	100%	100%	81%	99%	85%
**A15_Gt12(84)_Ps**	82%	100%	97%	100%	84%	82%	87%	100%	100%	81%	99%	77%
**Lpn-Thunderbay**	82%	100%	91%	100%	84%	79%	91%	100%	100%	93%	100%	85%

**L09-313_Cl_Gt84(116)_D**	82%	100%	91%	100%	84%	79%	91%	100%	100%	93%	100%	85%
**L01-443_Cl_Gt64(74)_D**	82%	100%	91%	100%	84%	79%	91%	100%	100%	93%	100%	85%
**A156_Gt64(74)_Ps**	82%	100%	91%	100%	84%	79%	91%	100%	100%	93%	100%	85%
**A129_Gt64(74)_Ps**	82%	100%	91%	100%	84%	79%	91%	100%	100%	93%	100%	85%
* Gene Identity												

Table S11: Summary of main plasmid features in *L. pneumophila* isolates H3, A129, A29, and L10_091.

Isolate designation	No. of base pairs (bp)	No. of CDS*	%GC
H3_Gt14(31)_D	103,432	111	40.0%
A129_Gt64(74)_Ps	72,682	78	38.9%
A29_Gt6(18)_Ps	25,160	27	38.6%
L10-091_Cl_Gt69(69)_D	23,296	25	38.6%

*CDS: Coding DNA Sequence

Table S12: Identification and distribution of prophages in the 55 *L. pneumophila* strains studied.														
**Strain designation**	**Replicon/Contig**	**Region**	**Region Length**	**Score**	**Specific Keyword**	**Region Position**	**No. tRNA**	**No. Total Proteins**	**No. Phage Hit proteins**	**No. Hypothetical Proteins**	**% Phages and Hypothetical Proteins**	**No. Bacterial Proteins**	**Attachement Site**	**% GC**
Lpn-LPE509	contig000001	1	26Kb	50	Plate, integrase and transposase	2,248,193 - 2,274,256	0	16	9	5	87.5	2	Yes	38.2
Lpn-Philadelphia1	contig000001	1	70.3Kb	70	Plate, integrase and transposase	1,158,151 - 1,188,905	0	26	13	10	88.4	3	Yes	37.2
Lpn-Thunderbay	contig000001	1	70.3Kb	70	Plate, integrase and transposase	1,165,464 - 1,196,215	0	26	13	10	88.4	4	Yes	37.2
Lpn-ATCC43290	contig000001	1	25.1Kb	60	Plate, integrase and transposase	1,118,202 - 1,143,394	0	29	15	11	89.6	3	yes	36.5
	contig000001	2	11.7Kb	70	head, integrase and transposase	2,316,998 - 2,328,793	0	11	7	3	90.9	1	Yes	37.2

Lpn-lpm7613	contig000001	1	11.7Kb	70	head, integrase and transposase	2,316,998 - 2,328,794	0	11	7	3	90.9	1	Yes	37.2
Lpn-Lens	contig000001	1	13.1Kb	30	integrase and plate	1,180,694 - 1,193,879	0	10	6	3	90	1	Yes	38.3
Lpn-Lorraine	contig000001	1	7.6Kb	10	NA	858,707 - 866,331	0	7	6	0	85.7	1	No	36.1
	contig000001	2	16.3Kb	20	integrase	1,107,057 - 1,123,388	0	7	4	0	57.1	3	Yes	37.5
	contig000001	3	22.7Kb	30	integrase and protease	2,553,434 - 2,576,203	1	9	7	1	88.8	1	Yes	40
	contig000001	4	31.9Kb	30	integrase	2,939,178 - 2,971,159	0	9	5	3	88.8	1	Yes	40.4
Lpn-HL06041035	contig000001	1	31.5Kb	50	Plate, and integrase	1,210,553 - 1,242,139	0	19	11	6	89.4	2	Yes	37.5
	contig000001	2	31.5Kb	70	Plate, and transposase	1,225,361 - 1,256,8	0	9	7	1	88.8	1	Yes	36.5

						64								
Lpn-D7631	contig000001	1	27.9Kb	40	Plate, integrase and transposase	1,192,648 - 1,220,587	0	16	9	7	93.7	1	Yes	38.6
Lpn-D7630	contig000001	1	27.9Kb	40	Plate, integrase and transposase	1,192,648 - 1,220,588	0	16	9	7	93.7	1	Yes	38.6
Lpn-D7632	contig000001	1	27.9Kb	40	Plate, integrase and transposase	1,192,648 - 1,220,589	0	16	9	7	93.7	1	Yes	38.6
Lpn-Paris	contig000001	1	18.7Kb	30	Plate and integrase	1,185,527 - 1,204,295	0	15	9	5	93.3	1	Yes	38.5
Lpn-OLDA	contig000001	1	18.7Kb	30	Plate and integrase	1,176,998 - 1,195,766	0	15	9	5	93.3	1	Yes	38.5
Lpn-Pontiac	contig000001	1	23.8Kb	30	Plate and integrase	1,214,034 - 1,237,858	0	13	8	4	92.3	1	Yes	37.8
Lpn-Toronto	contig000001	1	16.2Kb	60	Plate, integrase and transposa	1,189,658 - 1,214,9	0	10	6	2	80	2	Yes	35.5

					se	29								
Lpn-Alcoy 2300/99	contig000001	1	19.7Kb	30	Plate and integrase	1,280,061 - 1,299,823	0	12	7	4	91.6	1	Yes	37
Lpn-Corby	contig000001	1	19Kb	30	Plate and integrase	1,286,720 - 1,287,753	0	12	7	4	91.6	1	Yes	38.2
A156_Gt64(74)_Ps	contig000001	1	20.8Kb	40	Protease, integrase and transposase	222,459 - 243,352	1	17	9	4	76.4	4	Yes	37.4
	contig000002	2	14Kb	30	integrase and transposase	436,455 - 461,457	0	13	8	3	84.6	2	Yes	35.6
A129_Gt64(74)_Ps	contig000003	1	14Kb	30	integrase and Plate	728 - 14,800	0	12	7	4	91.6	1	Yes	38.4
A15_Gt12(84)_Ps		NA	NA	NA	NA	NA	NA	NA	NA	NA	NA	NA	NA	NA
A139_Gt4(17)_Ps		NA	NA	NA	NA	NA	NA	NA	NA	NA	NA	NA	NA	NA
A5_Gt4(17)_Ps	contig000001	1	7.6Kb	10	NA	9,003 - 16,627	0	7	6	0	85.7	1	No	36
A29_Gt6(18)_Ps	contig000008	1	19.5Kb	30	integrase and Plate	263,913 - 283,445	0	13	8	4	92.3	1	Yes	37.2

A131_Gt6(18)_Ps	contig000003	1	19.5Kb	30	integrase and Plate	263,913 - 283,445	0	13	8	4	92.3	1	Yes	36.3
	contig000004	2	35.9Kb	30	recombinase	79,639 - 115,554	0	9	6	1	77.7	2	Yes	37.2
A193_Gt40(47)_Ps	NA	NA	NA	NA	NA	NA	NA	NA	NA	NA	NA	NA	NA	NA
A194_Gt40(47)_Ps	contig000001	1	13.1Kb	30	integrase and Plate	1,144,101 - 1,157,286	0	10	6	3	90	1	Yes	38.3
A195_Gt40(47)_Ps	contig000002	1	9.7Kb	20	protease	65,660 - 75,400	1	7	6	1	100	0	no	39.6
A138_Gt9(92)_Ps	contig000007	1	20.3K	40	integrase and Plate	25,862 - 46,223	0	12	7	4	91.6	1	Yes	38.4
A112_Gt10(93)_Ps	NA	NA	NA	NA	NA	NA	NA	NA	NA	NA	NA	NA	NA	NA
A114_Gt10(93)_Ps	NA	NA	NA	NA	NA	NA	NA	NA	NA	NA	NA	NA	NA	NA
A127_Gt10(93)_Ps	NA	NA	NA	NA	NA	NA	NA	NA	NA	NA	NA	NA	NA	NA
A108_Gt10(93)_Ps	NA	NA	NA	NA	NA	NA	NA	NA	NA	NA	NA	NA	NA	NA
A166_Gt8(142)_Ps	NA	NA	NA	NA	NA	NA	NA	NA	NA	NA	NA	NA	NA	NA
H34_Gt22(102)_D	contig000002	1	7.7Kb	20	Plate	236,746 - 244,461	0	11	6	4	90.9	1	No	37.4
H29_Gt22(100)_D	contig000004	1	8.9Kb	30	Plate and integrase	307 - 9,269	0	12	7	4	91.6	1	No	37.7

H35_Gt22(102)_D	contig000007	1	8.9Kb	30	Plate and integrase	307 - 9,269	0	12	7	4	91.6	1	No	37.7
H39_Gt4(17)_D	contig000002	1	21Kb	30	Protease and integrase	53,825 - 74,871	1	9	7	1	88.8	1	Yes	39.9
H3_Gt14(31)_D	contig000001	1	19.1Kb	40	Plate and integrase	1,235,020 - 1,254,153	0	20	9	8	85	3	Yes	36.3
H23_Gt14(30)_D	NA	NA	NA	NA	NA	NA	NA	NA	NA	NA	NA	NA	NA	NA
H1_Gt14(31)_D	contig000001	1	13.1Kb	30	Plate and integrase	140,500 - 153,676	0	10	6	3	90	1	Yes	38.2
H2_Gt14(31)_D	contig000007	1	19.1Kb	40	Plate and integrase	432,357 - 451,502	0	20	9	8	85	3	Yes	36.3
L09-313_Cl_Gt84(116)_D	contig000005	1	7.7Kb	20	Plate	297,105 - 304,820	0	11	6	4	90.9	1	No	37.4
L01-443_Cl_Gt64(74)_D	contig000006	1	25Kb	40	Transposase and integrase	161,092 - 186,094	0	13	8	3	84.6	2	Yes	35.6
	contig000007	2	7.6Kb	10	NA	259,232 - 266,856	0	7	6	0	85.7	1	No	36.2
L10-091_Cl_Gt69(69)_D	contig000002	1	5.6Kb	30	Head	1,954 - 7,588	0	8	6	1	87.5	1	No	36.5
L12-317_Cl_Gt72(66)_D	contig000005	1	7.6Kb	10	NA	43,883 - 51,507	0	7	6	0	85.7	1	no	36.1

L11-209_Cl_Gt29(27)_D	NA	NA	NA	NA	NA	NA	NA	NA	NA	NA	NA	NA	NA	NA
L10-023_Cl_Gt75(49)_D	contig000001	1	23.8Kb	30	Plate and integrase	1,196,930 - 1,220,754	0	13	8	4	92.3	1	yes	37.8
L09-329_Cl_Gt75(49)_D	NA	NA	NA	NA	NA	NA	NA	NA	NA	NA	NA	NA	NA	NA
L02-521_Cl_Gt29(27)_D	NA	NA	NA	NA	NA	NA	NA	NA	NA	NA	NA	NA	NA	NA
L02-465_Cl_Gt27(133)_D	contig000007	1	7.6Kb	10	NA	1,196,930 - 1,220,754	0	7	6	0	85.7	1	No	36.2
L04-545_Cl_Gt40(47)_D	contig000007	1	23Kb	30	Plate and integrase	15,451 - 38,507	0	10	6	3	90	1	Yes	40.3
L06-153_Cl_Gt71(135)_D	contig000001	1	7.6Kb	10	NA	139,563 - 147,187	0	7	6	0	85.7	1	No	36
L06-129_Cl_Gt71(135)_D	contig000001	1	7.6Kb	10	NA	2,031 - 9,655	0	7	6	0	85.7	1	No	36
L04-041_Cl_Gt30(137)_D	contig000002	1	19.8Kb	20	integrase	70,310 - 90,170	0	14	6	5	78.5	3	Yes	36.6
	contig000003	2	7.7Kb	20	Plate	25,004 - 32,719	0	11	6	4	90.9	1	No	37.4

L05-341_Cl_Gt8(132)_D	NA	NA	NA	NA	NA	NA	NA	NA	NA	NA	NA	NA	NA	NA

NA: Not Available

Comparative genomics of *Legionella pneumophila* isolates from the West Bank and Germany support molecular epidemiology of Legionnaires' disease

Zayed et al.

SUPPLEMENTARY MATERIALS

FIGURES

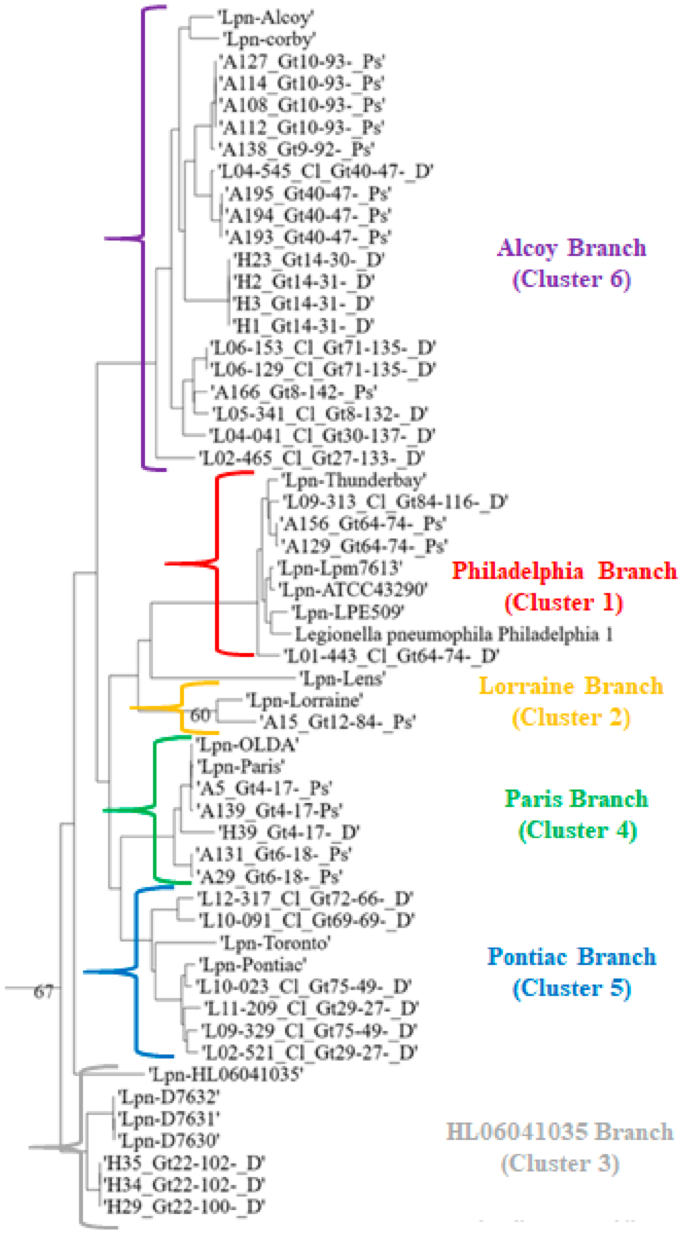

Figure S1. Phylogenomic tree of 55 *L. pneumophila* genomes based on the GBDP phylogenetic analyses retrieved from the TYGS website. The branch lengths are scaled in terms of GBDP distance formula d4. Color coding specific for each *L. pneumophila* branch

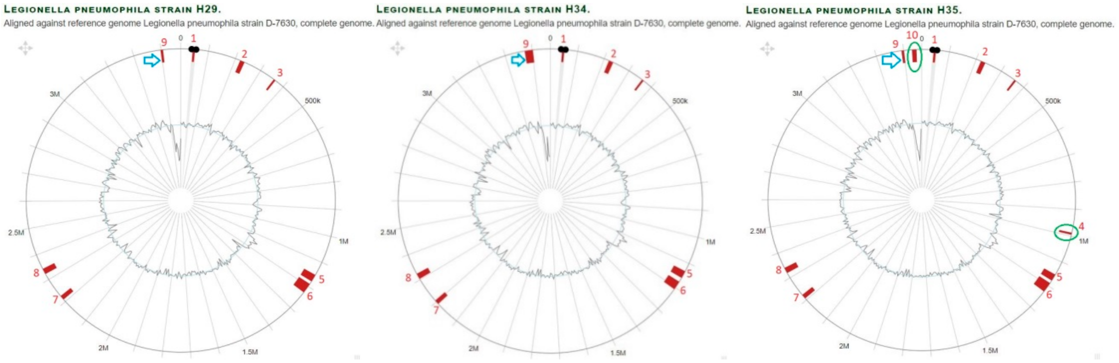

Figure S2: Comparison of Genomic islands in branch HL06041035 of *L. pneumophila* strain H29, H34 and H35 aligned against reference genome *L. pneumophila* strain HL06041035. The outer layers, the circle shows: (i) nucleotide positions in megabase pairs (Mbp) (black); (ii)Island Viewer-annotated potential genomic islands (GI) are labeled accordingly (red); (iii) Extra GIs (marked Green oval); (iv) GI different size (Blue arrow).

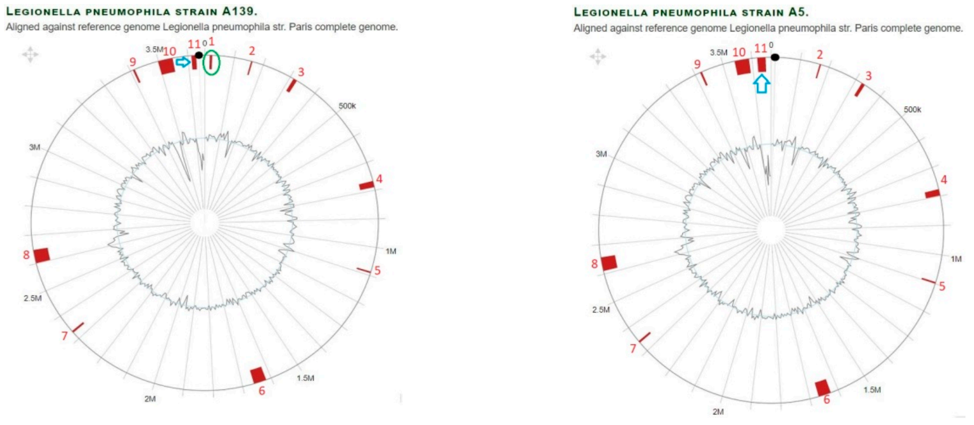

Figure S3: Comparison of Genomic islands in branch Paris of *L. pneumophila* strain A5 and A139 aligned against reference genome *L. pneumophila* strain Paris. The outer layers, the circle shows: (i) nucleotide positions in megabase pairs (Mbp) (black); (ii)Island Viewer-annotated potential genomic islands (GI) are labeled accordingly (red); (iii) Extra GIs (marked Green oval); (iv) GI different size (Blue arrow).

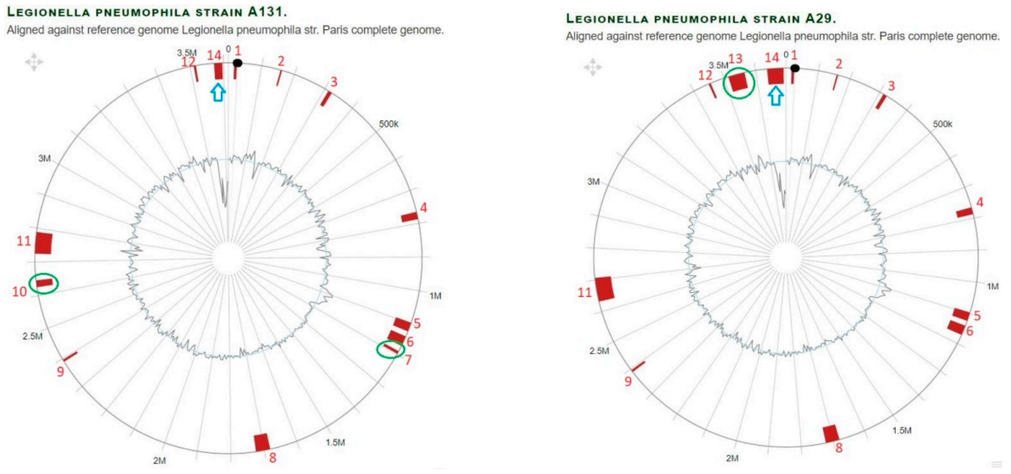

Figure S4: Comparison of Genomic islands in Branch Paris of *L. pneumophila* strain A29 and A131 aligned against reference genome *L. pneumophila* strain Paris. The outer layers, the circle shows: (i) nucleotide positions in megabase pairs (Mbp) (black); (ii)Island Viewer-annotated potential genomic islands (GI) are labeled accordingly (red); (iii) Extra GIs (marked Green oval); (iv) GI different size (Blue arrow).
